# Diagnostic accuracy of physician-staffed emergency medical teams: a retrospective observational cohort study of prehospital versus hospital diagnosis in a 10-year interval

**DOI:** 10.1186/s13049-019-0617-3

**Published:** 2019-04-02

**Authors:** Jens-Christian Schewe, Jochen Kappler, Katharina Dovermann, Ingo Graeff, Stefan Felix Ehrentraut, Ulrich Heister, Andreas Hoeft, Stefan Ulrich Weber, Stefan Muenster

**Affiliations:** 1 Department of Anesthesiology and Critical Care Medicine, University Hospital of Bonn, Sigmund-Freud-Strasse 25, 53127 Bonn, Germany; 2 Department of Emergency Medicine, Bonn, University Hospital Bonn, Bonn, Germany; 3 Emergency Medical Service Bonn, Bonn, Germany; 4 Department of Anesthesiology, Critical Care and Pain Medicine, Heilig Geist Krankenhaus, Cologne, Germany

**Keywords:** Diagnostic accuracy, Physician-staffed medical emergency teams, Prehospital emergency care

## Abstract

**Background:**

In Germany, emergency medical teams are staffed with physicians but evidence regarding their prehospital diagnostic accuracy remains poor.

**Objective:**

To evaluate the out-of-hospital diagnostic accuracy of physician-staffed emergency medical teams (PEMTs).

**Methods:**

A retrospective observational cohort study involving the Emergency Medical Service Bonn, Germany, from January to December 2004 and 2014 respectively. A total of 8346 patients underwent medical treatment by PEMTs, of which 1960 adult patients (inclusion criteria: ≥18 years of age, hospital diagnosis available) were included for further analysis. Reasons for non-inclusion: death on scene, outpatient, interhospital transfer, mental illness, false alarm, no hospital medical history available. The overall diagnostic accuracy (correct or false) of PEMTs was measured after matching the prehospital diagnosis with the corresponding diagnosis of the hospital. Secondary outcome measures were incidence of common PEMT diagnoses (acute coronary syndrome (ACS), dyspnea, stroke/intracerebral bleeding), recognition rate of a given disease by PEMTs, and prehospital diagnostic accuracy in elderly patients.

**Results:**

PEMT calls increased 2-fold over a decade (2004: *n* = 3151 vs. 2014: *n* = 5195). Overall diagnostic accuracy of PEMTs increased from 87.5% in 2004 to 92.6% in the year 2014. The incidence of common PEMT diagnoses such as ACS, dyspnea or stroke/intracerebral bleeding increased 2-fold from 2004 to 2014. The recognition rate of a given disease by the PEMT varied between 2004 and 2014: an increase was observed when a stroke/intracerebral bleeding was diagnosed (2004: 67% vs. 2014: 83%; *p* = 0.054), a decreased rate of recognition occurred when a syncope/collapse was diagnosed (2004: 81% vs. 2014: 56%; *p* = 0.007) and a sepsis appears to be a rare event for EMS personnel (2004: 0% vs. 2014: 23%). Linear regression analysis revealed that the prehospital diagnostic accuracy decreases in the elderly patient.

**Conclusions:**

The overall prehospital diagnostic accuracy of PEMTs improved between the year 2004 and 2014 respectively. Our findings suggest that the incidence of common diseases (ACS, dyspnea stroke/intracerebral bleeding, sepsis) increased over a 10-year period. Diagnostic accuracy of different diseases varied but generally decreased in the elderly patient. Regular training of EMS personnel and public campaigns should be implemented to improve the diagnostic accuracy in the future.

**Electronic supplementary material:**

The online version of this article (10.1186/s13049-019-0617-3) contains supplementary material, which is available to authorized users.

## Introduction

In Germany, physician-staffed Emergency Medical Teams (PEMT) are part of the local Emergency Medical Service (EMS) [1]. These teams respond to calls of high urgency to perform prehospital emergency care. This is important for various diseases such as acute coronary syndrome (ACS), stroke, severe respiratory failure or others where a medical intervention carried-out by a physician should be performed immediately on scene. Physicians, as opposed to paramedics, provide a diagnosis once they have examined the patient. This offers the opportunity to admit the patient to a dedicated hospital with the given medical specialty for the disease. This is important, as the number of hospitals in Germany have decreased within the last decade [2]. Lastly, economic reasons force hospitals to turn into special care centers. As a consequence, some medical disciplines are not available. In this context, the existence of a diagnosis allows a straight forward admission to the right hospital.

Evidence with regard to the diagnostic accuracy of emergency physicians in the field remains poor [3–5]. In 1997, Arntz and co-workers investigated in a German retrospective cohort study the diagnostic accuracy of emergency physicians by comparing the pre-hospital diagnosis with the hospital diagnosis [5]. In 10% of all cases, physicians failed to correctly diagnose the underlying diseases either by under- or overestimating the medical course of the disease. The authors found the failure rate to be even higher in certain subgroups such as stroke (14%) or severe respiratory failure (13%). Another population-based registry analysis reported trend data regarding the EMS diagnoses but without evaluating the diagnostic accuracy of the participating PEMTs [4]. More recent trials are missing to mirror the current situation and a possible improvement in EMS with PEMTs.

Here, we seek to evaluate the overall out-of-hospital diagnostic accuracy of PEMTs in comparison of the year 2004 to 2014. Because the EMS of Bonn has grown over the last years, we hypothesized that the inquiries of emergency calls would (1) increase over a 10-year interval, and that (2) the incidence of a given disease which is seen by the PEMTs would have changed. We further investigated (3) whether the diagnoses of the emergency physician were correct or false, and (4) whether the patient’s age may be relevant in the accuracy of a prehospital diagnosis.

## Methods

### Study design

This retrospective observational cohort study was operated in compliance with the Strengthening the Reporting of Observational Studies in Epidemiology statement (STROBE statement). Ethical approval for this study was provided by the Ethic Committee (N° 054/04) of the University Hospital Bonn, Germany on February 14th, 2005 and the need for informed consent was waived.

### Setting

The EMS Bonn serves approximately 320,000 residents in an area of 141 km^2^. A total of 12 ambulances and 3 PEMTs perform medical treatment in a double-response system. Ambulances are staffed with an emergency medical technician and a paramedic. PEMTs consists of a paramedic and a physician trained in emergency care. Physicians are licensed to practice emergency care according to the German Medical Academy. PEMTs are established in the EMS of Bonn city since 1971. Data from PEMT interventions was collected at the EMS station with the highest volume of PEMT calls. Here, 2 out of 3 PEMTs are located and physicians are anesthesiologists from the university hospital. The EMS responds to 33,000 emergency calls annually, including approximately 10,000 PEMT interventions. A total of 8 hospitals with emergency departments operate day and night to admit patients from the EMS. In this retrospective observational study, all adult patients were recruited who underwent medical treatment by an PEMT in the EMS of Bonn between January and December 2004 or 2014 respectively.

### Participants

To evaluate the diagnostic accuracy of PEMTs, a retrospective, record-based review was performed in all adult patients (inclusion criteria: ≥18 years of age, out-of-hospital diagnosis available) who underwent medical treatment by an PEMT in 2004 or 2014 respectively. Data routinely recorded by the PEMT (such as age, sex, diagnosis, time point of treatment) was extracted, anonymized and transferred to a comprehensive database to identify eligible patients. Diagnoses were encoded according to the International Statistical Classification Of Disease And Related Health Problems, 10th revision, German Modification (ICD-10-GM) which is the official classification system in Germany to encode diseases in both inpatient and outpatient medical care. For the eligible cohort, patients were excluded from the analysis when they died on scene (no hospital medical history available), underwent outpatient treatment (no admission to hospital), when an interhospital transfer was performed and overseen by the PMET, when a false alarm of the unit occurred, and in cases of mental illness (hospital medical history unavailable due to increased measures of protected health information by national law).

### Variables

To create a ranking and incidence of the 20 most common PEMT diagnoses, all eligible PEMT interventions (2004: *n* = 2572 and 2014: *n* = 4276, respectively) were analyzed. When the hospital medical history of a patient was available, it was matched with the out-of-hospital PEMT records of the same patient to measure the recognition rate of a given PEMT diagnosis. The out-of-hospital PEMT protocol was matched with the corresponding hospital medical history of the individual patient to investigate the diagnostic accuracy of the PEMT diagnosis. The discrimination and evaluation in correct and false was independently performed by three experienced emergency physicians. In case of disparate evaluations, a final result was obtained by majority decision. Beside their certification as emergency physicians (licensed by the German Medical Board) all of the three physicians are board-certified anesthesiologists. In addition, all of the three physicians passed a two-year fellowship in intensive care medicine and are certified as specialists in the field of critical care by the German Medical Board.

### Statistical analysis

All data are expressed either as absolute or percent values. Microsoft Excel 2017 (Microsoft Corporation, Redmond, WA, USA) was used to create the comprehensive database. Statistical analyses were performed using IBM SPSS Statistics for Windows, Version 20.0 (IBM Corporation, Armonk, NY, USA). In Fig. 3, variables were tested for normality using the Shapiro-Wilk test and comparisons within a given diagnosis were performed using the Mann-Whitney-U-test. *P*-values less than 0.05 were considered significant. In Fig. 4, a 95% confidence interval was calculated and variables were tested for significance with a Chi-square test. *P*-values less than 0.05 were considered significant. In Fig. 5, linear regression analyses were performed to investigate whether or not there were correlations between diagnostic accuracy and the patient’s age.

## Results

### PEMT calls increased 2-fold and the overall diagnostic accuracy improved over a 10-year interval

**Fig. 1:**
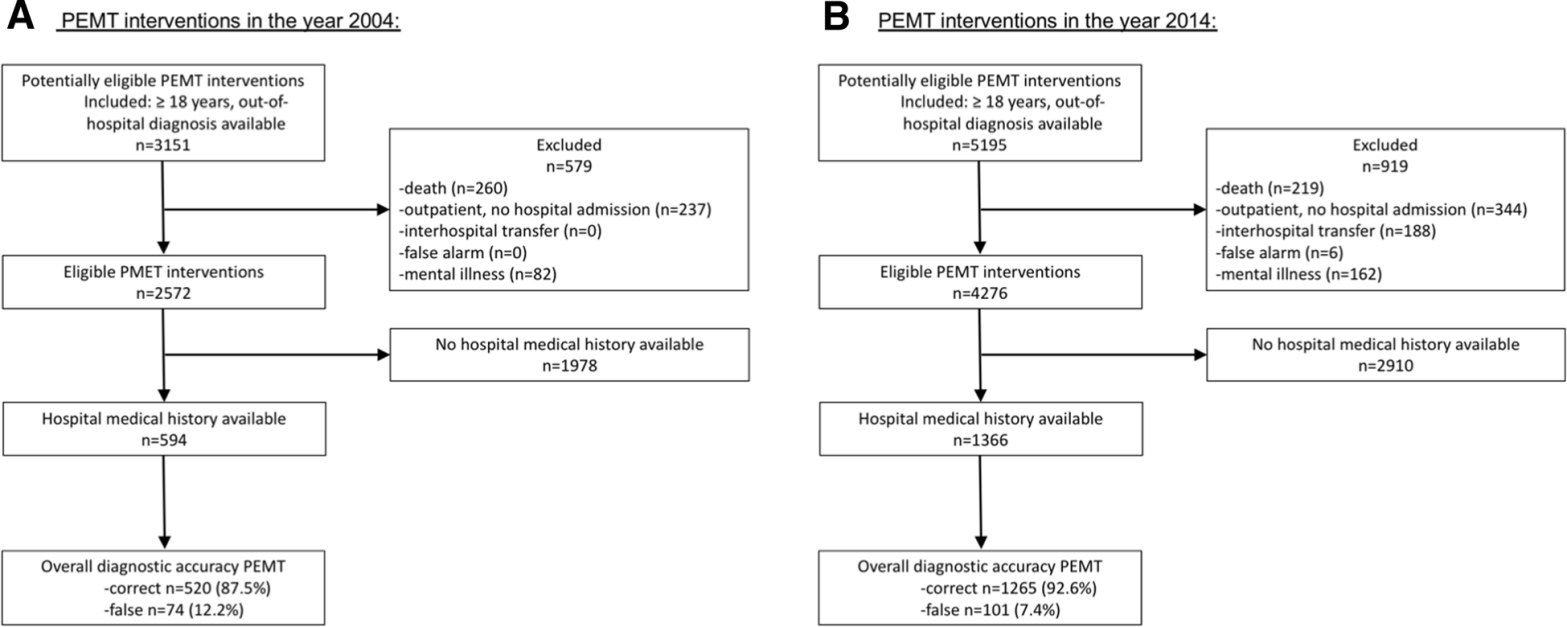
Flow diagram with inclusion and exclusion criteria of the eligible cohort. The number of emergency calls increased between 2004 (**a**) and 2014 (**b**)

In 2014, the number of PEMT dispatches increased nearly 2-fold when compared to the year 2014 (Fig. 1 a and b). Medical histories from the hospitals are routinely requested by the EMS for internal quality measures on a voluntary base but the response rate varies. In 2004, medical histories were obtained in 594 out of 2572 cases whereas in 2014 medical records were received in 1366 out of 4276 patients. When the prehospital diagnosis of the PEMT was matched with the hospital diagnosis, the overall diagnostic accuracy of the emergency physicians improved between 2004 when compared to 2014 (Figs. 1a, b and 4a, respectively).

### Incidence of the 20 most common PEMT diagnoses and their ranking over a 10-year interval

Because the inquiries of PEMT dispatches increased over a decade, we investigated whether the increase of emergency calls is related to an elevated incidence of a given disease. We calculated the incidence of certain diseases per 100,000 residents per year.

**Fig. 2:**
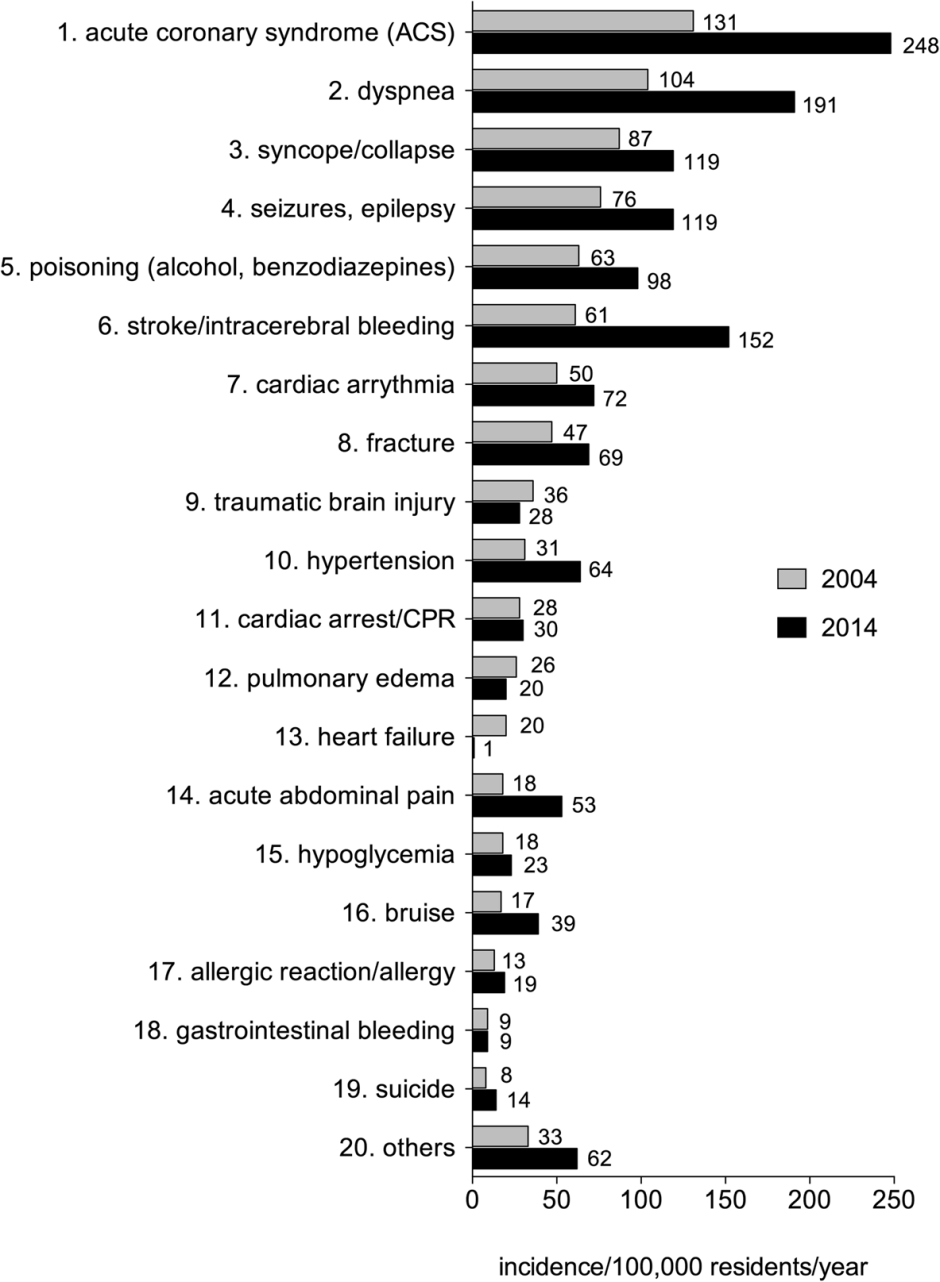
Comparison of the incidence of a given disease per 100,000 residents per year. Grey bars represent the incidences in the year 2004 whereas black bars represent the values of the year 2014. Numbers beside the bars represent the incidence. Ranking of the 20 most common diseases in the year 2004 is listed on the y-axis and ordered by numbers

The incidence of the ACS or dyspnea increased 2-fold from 2004 to 2014 whereas the PEMT intervention stroke/intracerebral bleeding showed a 2.5-fold increase when compared to 2004 (Fig. 2). In general, many of the top 20 PEMT diagnoses showed at least a moderate elevated incidence in 2014 when compared to 2004. These data demonstrate that the increase in PEMT calls is related to an overall elevated incidence of the 20 most common PEMT diagnoses.

### Diagnostic accuracy of the PEMT

The afore mentioned increase of PEMT diagnoses such as the ACS, dyspnea or stroke/intracerebral bleeding might be related to an improved recognition rate of the PEMTs. Therefore, we intended to measure the recognition rate of a given disease by the PEMT in the prehospital setting. We matched the medical history of the admitting hospital with the corresponding PEMT protocol.

**Fig. 3:**
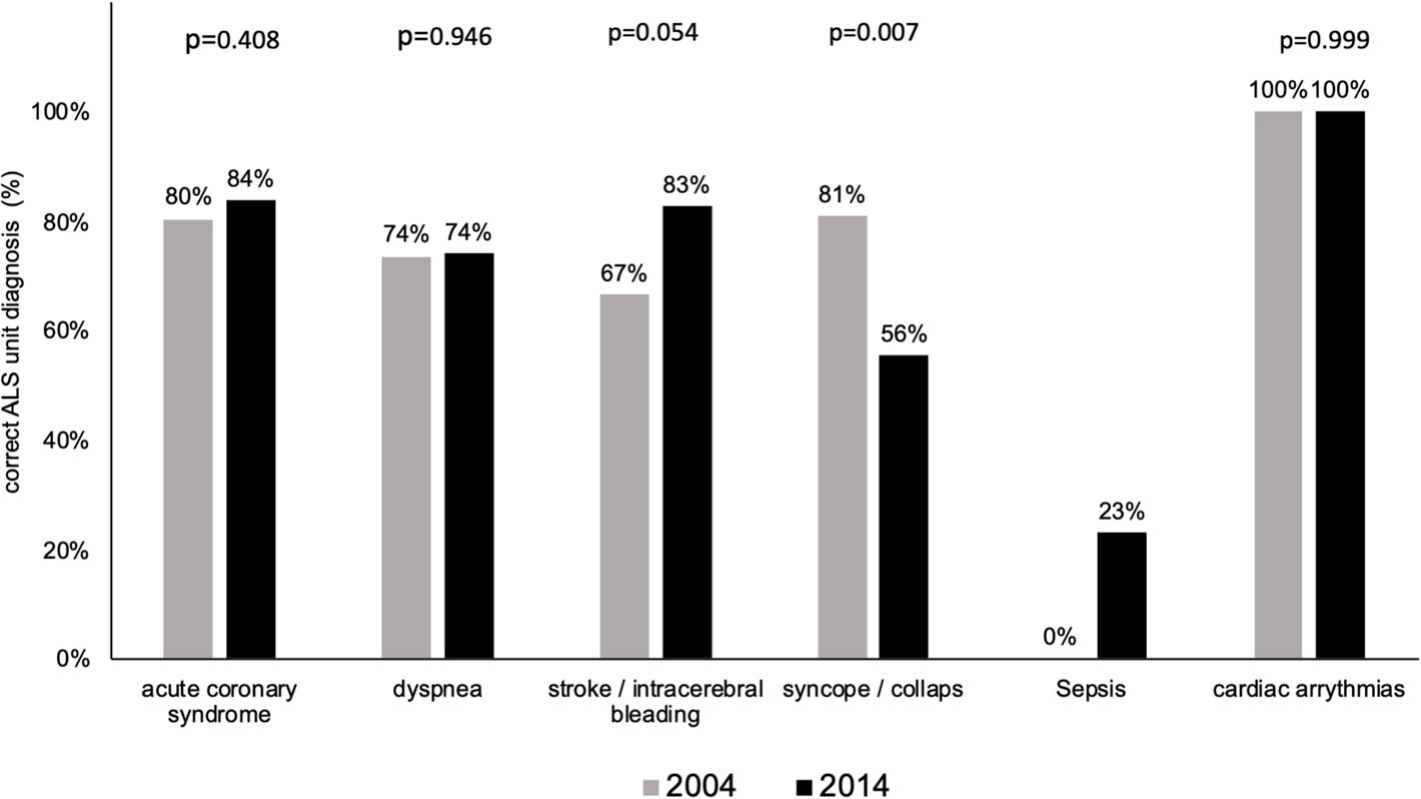
Correct PEMT diagnoses of a given disease when the medical history of the hospital has been matched with PEMT diagnoses. Values on the y-axis represent the percentage of correct PEMT diagnoses. Diseases are plotted on the x-axis. Grey bars represent the year 2004 and black bars the year 2014 respectively

When an ACS occurred, PEMTs detected the disease with the same rate in 2004 as in 2014 (Fig. 3). Dyspnea was correctly diagnosed by the PEMT in 74% in 2004 when compared to the hospital medical history and did not change in 2014 (Fig. 3). In 2004, a stroke or intracerebral bleeding was detected less often by the PEMT when compared to the year 2014, hence statistical analysis did not reach significance. In contrast, the recognition rate of a syncope or a collapse decreased in 2014 when compared to the year 2004 (Fig. 3). In 2004, sepsis has not been correctly identified by the PEMTs. In 2014, this disease was diagnosed in 23% correctly of all sepsis cases. The recognition rate of cardiac arrhythmias occurred to be 100% correct in both 2004 and 2014 respectively.

Once the PEMT obtained a prehospital diagnosis in the field, we investigated whether their diagnosis was correct or false. We compared whether the diagnosis obtained by the PEMT matched the medical history of the hospital.

**Fig. 4:**
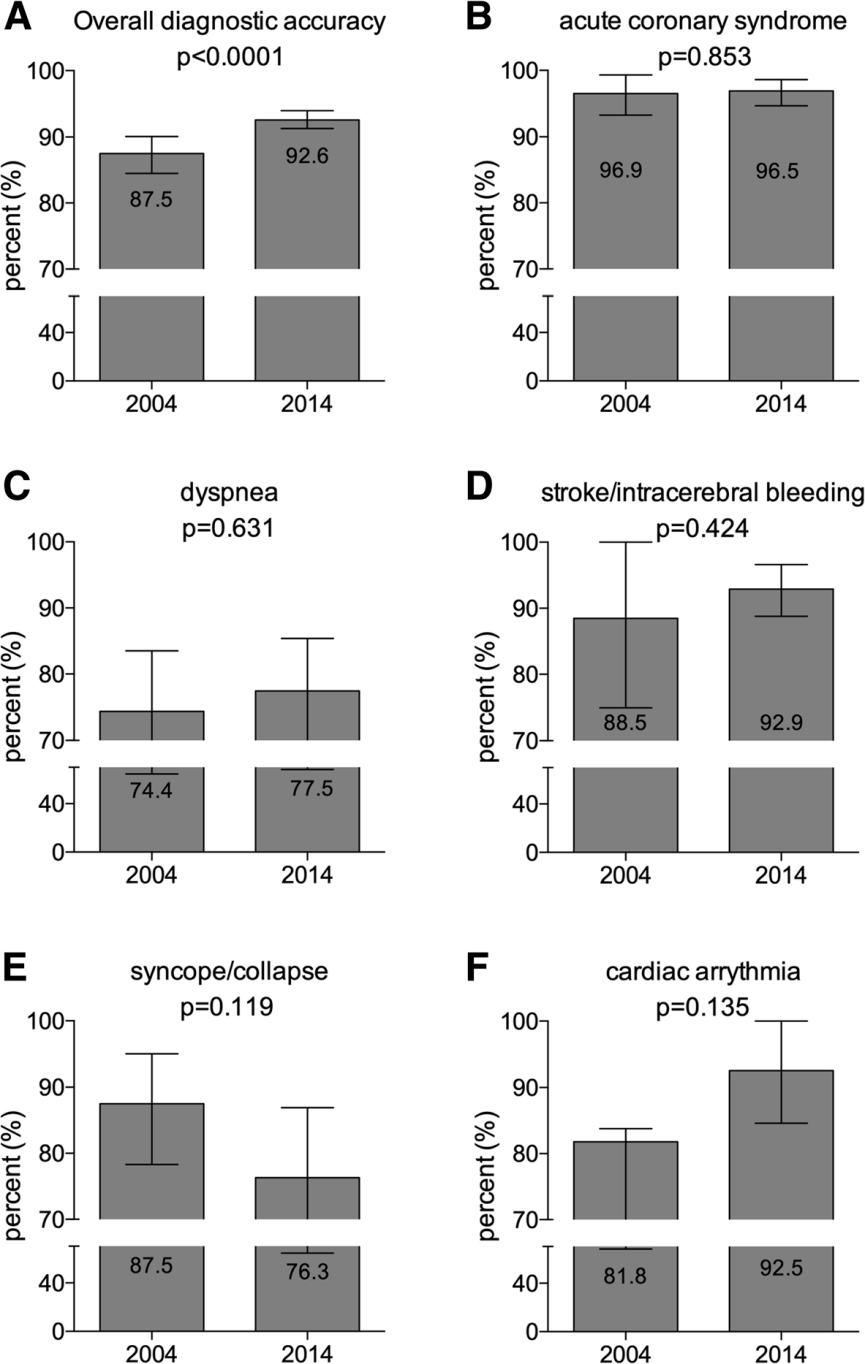
Diagnostic accuracy of PEMTs in the year 2004 and 2014. Here, the PEMT protocol was matched with medical history of the hospital. Panel (**a**) represents the overall diagnostic accuracy where as panels (**b**) to (**f**) represent specific diseases. Bars represent the 95% confidence intervals. On each graph the left grey columns represent the year 2004 whereas the right grey columns represent the year 2014. Values within the columns are the percent of diagnostic accuracy of the given disease

The overall diagnostic accuracy increased between 2004 and 2014 (Fig. 4 a). ACS, dyspnea, syncope/collapse, stroke/intracerebral bleeding, and cardiac arrhythmias were among the most common disease states that were seen by PEMT and were therefore chosen for a more detailed analysis. In 2004, the majority of ACS were correctly diagnosed by the PEMT and there was no change when compared to the year 2014 (Fig. 4 b). In terms of dyspnea, the diagnostic accuracy of the PEMT did not improve between 2004 and 2014 (Fig. 4 c). In 2014, PEMTs improved their hit rate in identifying a stroke/intracerebral bleeding when compared to 2004, hence changes are not statistically significant (Fig. 4 d). In terms of the diagnosis syncope/collapse, the majority of cases were correctly identified in 2004 and 2014, hence differences were not statistically significant (Fig. 4 e). The diagnostic accuracy of cardiac arrhythmias increased between 2004 and 2014, but differences were also not statistically significant (Fig. 4 f).

### Diagnostic accuracy of the PEMT decreases in the elderly patient

To further investigate whether the patient’s age may have an impact on the diagnosis accuracy we correlated the rate of false diagnoses in relation to the patient’s age.

**Fig. 5:**
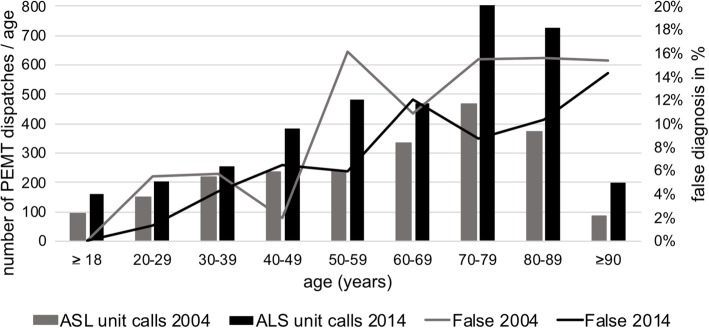
Diagnostic accuracy of the PEMTs versus age group of the patients. Grey columns represent the number of PEMT dispatches per age group in the year 2004 and black columns represent the number of PEMT dispatches per age group in the year 2014. Grey lines express the percent of false diagnoses per age group in the year 2004 whereas black lines express the percent of false diagnoses per age group in the year 2014. Linear regression analysis was performed to measure a correlation between diagnostic accuracy and the patient’s age

In the year 2004, the rate of false diagnoses increased with age up to 15% starting at a patient age of 60 years and older (Fig. 5, r^2^ = 0.7298). In 2014, false diagnoses of PEMTs were found up to a rate of 18% in patients that were older than 60 years (Fig. 5, r^2^ = 0.8852).

### Diagnostic accuracy of the PEMT does not vary over the 24 h-shift of EMS personnel

Please see Additional file 1: Figure S1 for more details.

## Discussion

In this study, we demonstrated that the diagnostic accuracy of PEMTs improved between 2004 and 2014. We further reported that the incidence of the ACS, dyspnea and stroke/intracerebral bleeding increased within 10 years. A decrease in diagnostic accuracy was demonstrated for the elderly patient.

In Europe, the number of emergency calls to EMS increased at least two-fold over the last 20 years [4, 6, 7]. We found similar results with a 39% increase in PEMT dispatches between 2004 and 2014. Reasons might be the growth and aging of population, a limited access to primary care physicians, and improved awareness of certain health issues through the implementation of campaigns on stroke or myocardial infarction [4].

A growing incidence for cardio-vascular, respiratory or thrombo-embolic pathologies has been reported in EMS systems [4, 8]. We report increased incidences for the most common 20 PEMT diagnoses. In 2014, the incidence of ACS (ranking first on our incidence scale) showed a two-fold increase when compared to 2004. One reason could be improved diagnostic options such as the implementation of 12-lead ECG devices on both PEMT units and ambulances [8–10]. Other reasons include a growing public awareness of myocardial infarction and specific training of EMS personnel [11]. Dyspnea was ranking second and we observed a 2.5-fold increase between 2004 and 2014. A similar trend was reported towards a growing incidence of respiratory distress in prehospital emergency care [4]. Acute dyspnea is more of a symptom rather than a diagnosis of a given disease state. Common diseases with acute dyspnea include ACS, decompensated congestive heart failure (often complicated by pleural effusion), and chronic obstructive pulmonary disease [12]. There is increasing evidence, that additional diagnostic tools such as the prehospital chest emergency ultrasound may serve as a new tool to facilitate additional therapeutic consequences in patients with respiratory distress and thereby possibly improves prehospital emergency care in those patients [12, 13]. However, a portable ultrasound device has not yet been implemented in the EMS of Bonn. In this case, the 2.5-fold increase of the diagnosis dyspnea is not related to a possible improvement by an additional diagnostic device. We also observed a 2.5-fold increase of the diagnoses stroke or intracerebral bleeding in emergency patients. A possible explanation could be an improved connection between public awareness, prehospital care and in-hospital treatment protocols [14]. The early recognition of stroke symptoms is crucial to minimize the delay of a life-(and brain-) saving recanalization therapy [14]. In general, public awareness of stroke symptoms still remains poor, with reported decision rates to call for an ambulance of less than 50% within the first hour of stroke symptoms [15]. Almost half of the stroke patients do not use an EMS at all to be admitted to the hospital [16]. Campaigns such as the FAST (F = facial paresis; A = arm drift; S = speech; T = time sensitivity) have been implemented to improve public awareness and evidence suggests an improvement in early stroke recognition [17, 18]. As this campaign has also been implemented in our city between 2004 and 2014, our data supports the afore mentioned hypothesis that public awareness of stroke symptoms might be improved by those campaigns. Also, prehospital emergency care is crucial when a stroke occurred to rapidly recognize the disease and to minimize the untreated downtime until a thrombolysis can be performed [19].

We intended to measure the diagnostic accuracy of PEMTs and investigated whether the increasing incidence of ACS, dyspnea or stroke/intracerebral bleeding might be related to an improved detection rate of a given disease. We found an increased rate of correct PEMT diagnoses when ACS or a stroke/intracerebral bleeding occurred. Statistical analysis revealed a *p*-value of *p* = 0.054, and therefore did not reach statistical significance hence the result could be validated as a trend towards statistical significance. As mentioned earlier, the improved availability of diagnostic tools such as the 12-lead ECG or the FAST algorithm for a stroke may have led to a better screening for these patients. In addition, regular and repeated training sessions with the focus on ACS, stroke and CPR of the EMS personnel possibly contributed to the increased diagnostic rate of these life-threatening diseases. Lastly, the increasing number of PEMT dispatches might have led to an improved performance of the individual emergency physician. Further studies are needed to prove this hypothesis. We found a higher incidence of syncope/collapse yet we observed a decrease in diagnostic accuracy of the PEMT diagnoses syncope/collapse. It might be that cases which have been diagnosed as syncope/collapse in 2004 have now been assigned as either ACS or stroke in 2014. On another note, the recognition rate of patients with sepsis increased by the year 2014. We did not observe any patient with a diagnosis of sepsis in the PEMT protocols in 2004 whereas these cases were found in 2014. The diagnostic accuracy of sepsis remains poor (23% correct PEMT diagnosis). For an individual emergency physician, sepsis itself remains a rare diagnosis with a total of 7 out of 1265 cases in 2014. We assume that sepsis (as an accompanying clinical sign for pneumonia, an urinary tract infection or other infectious diseases) occurs in numerous cases but remains undefined as a disease itself by the PEMTs. A large retrospective trial reported that sepsis occurs more often than a myocardial infarction or a stroke in prehospital emergency care [20]. Also, the majority of sepsis cases remained undetected until the disease was diagnosed at hospital admission [20]. Partly, because the diagnostic tools to clarify if a sepsis is present (WBC count, blood cultures) are incomplete. Recently, diagnostic tools such as the quick SOFA score have been suggested to screen for sepsis patients but their diagnostic value for EMS personnel needs to be determined [21]. This is important, as patients who have been diagnosed by EMS personnel with sepsis had a shorter time to both antibiotics and early-goal directed therapy initiation at the admitting hospital [22].

We further evaluated whether the diagnostic accuracy of the PEMTs depends on the daytime and the shift-length when the emergency call was requested. We did not find any differences. Results from other studies are inconsistent. Guyette and co-workers reported no changes in cognitive performance when EMS workers worked in either 12 h- or 24 h-shifts [23]. In contrast, Weaver and colleagues found an increasing risk for occupational injuries with extended shift-length as a marker for a reduced cognitive performance [24].

Large clinical trials demonstrate that elderly patients aging 80–89 years have an increasing need for emergency care with a predominance of medical issues of non-traumatic origin [4, 25]. We found similar results with peaks of emergency calls in the age group 70–90 years. Some authors linked this increase to demographic changes and less access to primary care physicians [4, 26]. We found that age is linked to the diagnostic accuracy of PEMTs peaking at the age group of 80–89 where the rate of false diagnoses was up to 15%. In our opinion, education and training of EMS personnel should start to focus more on geriatric patients as the majority of emergency requests are seen in the elderly. Geriatric patients often suffer from several diseases and therefore the on-scene-symptoms can be vague or a composition of themselves. In terms of a diagnosis-based referral to the hospital, the emergency physician might tend to make a quick diagnosis to enforce a hospital admission.

A major limitation of the study is the low rate of hospital medical histories that were available for further analyses (23 and 32% respectively). This is due to the fact that hospital medical records were received on a voluntary basis as part of internal quality measures. As this study is based on an observational design, our findings relay on the given documentation. This implies a considerable risk of selection bias. A larger number of included cases would likely narrow our findings and show more significant results. Another limitation of this study is the retrospective design and as opposed to hospitals, the diagnostic opportunities of EMS are naturally limited and based on the skills of single EMS-teams. Some of the prehospital diagnoses are rather a symptom than a final diagnosis (“dyspnea, syncope/collapse”) but the only diagnostic data that was achievable in this prehospital emergency care study. The group of emergency physicians was homogeneous as only anesthesiologists provided prehospital emergency care.

In summary, we investigated the diagnostic accuracy of PEMTs by comparing the prehospital diagnosis with the medical history of the hospital over a 10-year interval. We found an increasing number of emergency calls, higher incidences of common emergency diseases and an improved overall diagnostic accuracy rate. Our data suggests that sepsis is still rarely diagnosed by the emergency care personnel. Elderly patients represent the majority of emergency requests but diagnostic accuracy of PEMTs decreases with the patient’s age. We did not find any influence of the daytime and the shift-length when the emergency call was requested on the diagnostic accuracy.

## Additional file

Additional file 1: **Figure S1.** Diagnostic accuracy of the PEMTs versus daytime when the emergency call is requested. Panel A represents the year 2004 in grey bars and lines, Panel B represents the year 2014 in black bars and lines. Linear regression analysis was performed to measure a correlation between diagnostic accuracy and daytime when the emergency call has been requested. X-axes represent the daytime hours beginning with start of shift (8 a.m.) and covering a 24 h length of shift. (DOCX 7507 kb)

## Abbreviations

ACS: acute coronary syndrome
CPR: cardiopulmonary resuscitation
EMS: emergency medical services
PEMTs: physician-staffed emergency medical teams
WBC: white blood cells
