# Supplementary material for: Diagnostic accuracy of physician-staffed emergency medical teams: a retrospective observational cohort study of prehospital versus hospital diagnosis in a 10-year interval
Source: Scand J Trauma Resusc Emerg Med. 2019 Apr 2;27:36. doi: 10.1186/s13049-019-0617-3 (PMC6446382; doi:10.1186/s13049-019-0617-3)
Supplement: Supplementary file 1 — Figure S1. Diagnostic accuracy of the PEMTs versus daytime when the emergency call is requested. Panel A represents the year 2004 in grey bars and lines, Panel B represents the year 2014 in black bars and lines. Linear regression analysis was performed to measure a correlation between diagnostic accuracy and daytime when the emergency call has been requested. X-axes represent the daytime hours beginning with start of shift (8 a.m.) and covering a 24 h length of shift. (DOCX 7507 kb) [file 13049_2019_617_MOESM1_ESM.docx]

**SUPPLEMENTAL MATERIAL**

**Diagnostic Accuracy Of Physician-Staffed Emergency Medical Teams: A Retrospective Observational Cohort Study Of Prehospital Versus Hospital Diagnosis in A 10-Year Interval**

Jens-Christian Schewe^1a^, Jochen Kappler^1a^, Katharina Dovermann^1^, Ingo Graeff^1,2^, Stefan Felix Ehrentraut^1^, Ulrich Heister^1,3^, Andreas Hoeft^1^, Stefan Ulrich Weber^4^, and Stefan Muenster^1^

^1^*Department of Anesthesiology and Critical Care Medicine, University Hospital Bonn, Bonn, Germany*

^2^*Department of Emergency Medicine, Bonn, University Hospital Bonn, Bonn, Germany*

^3^*Emergency Medical Service Bonn, Bonn, Germany*

^4^*Department of Anesthesiology, Critical Care and Pain Medicine, Heilig Geist Krankenhaus, Cologne, Germany*

**Supplemental Results:**

**Diagnostic accuracy of the PEMT does not vary over the 24h-shift of EMS personnel**

PEMTs frequently operate in 24h-shifts. As the human factor may influence the diagnostic accuracy, we intended to measure whether the daytime and the shift length may have an impact on the recognition rate of the PEMT.

Linear regression analysis did not show any correlation between the shift length and the rate of false diagnoses neither in 2004 nor in 2014 over the period of 24h (Figure S1).

These data suggest that long-lasting working shifts for up to 24h do not have an impact on the overall diagnostic accuracy of PEMTs.

**
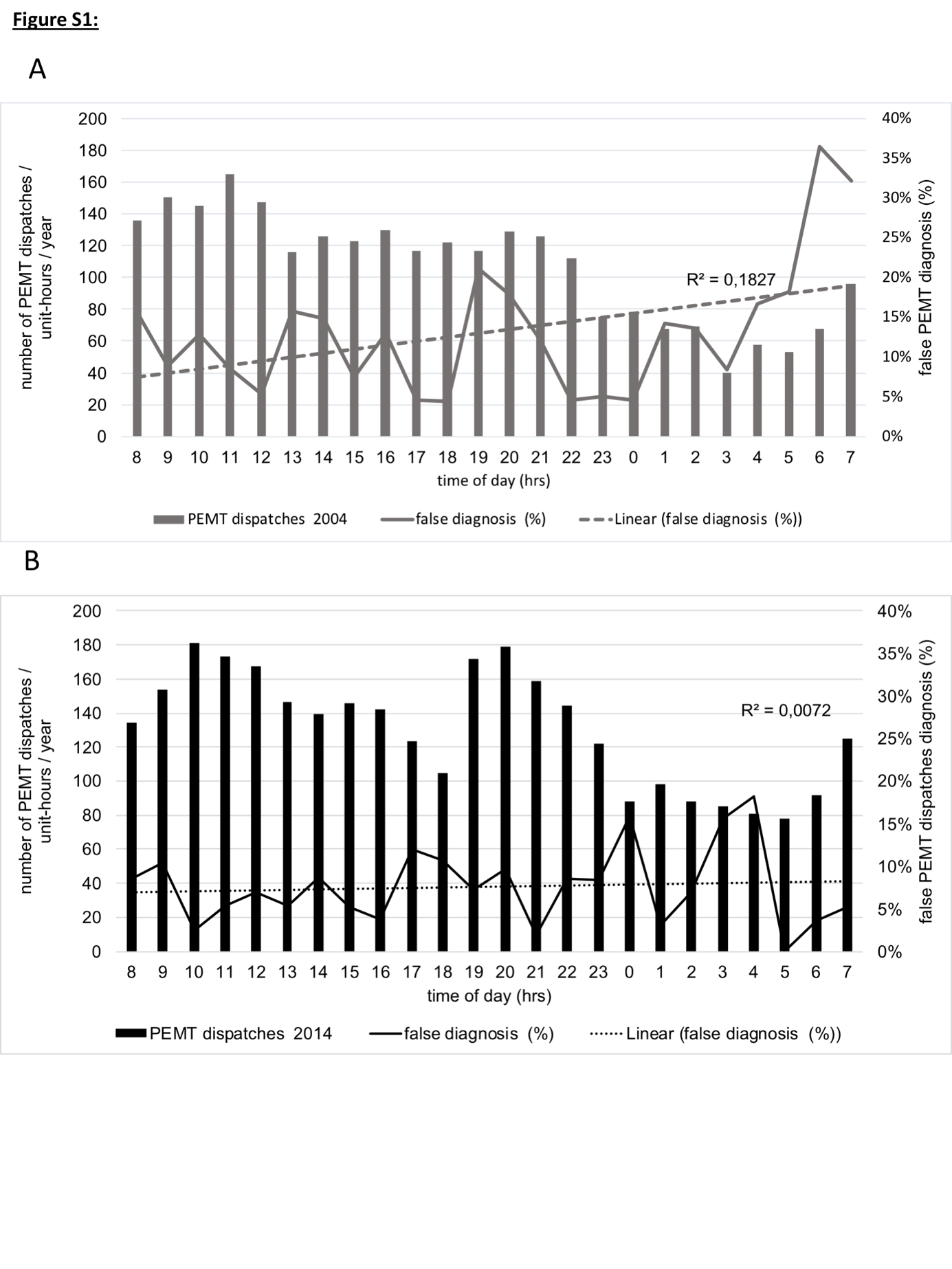
**
